# Health Tract, No. 184

**Published:** 1864-02

**Authors:** 


HEALTH TRACT, No. 184.

### KINDNESS REWARDED.

It is a dreadful thing to be old and poor, and have no home; but there is a deeper depth of human calamity than this—it is to have, in addition, an old age of wasting, wearing sickness, which is often superinduced by that constant depression of mind which attends the consciousness of being alone and friendless and in want. One of the very best means of avoiding an old age of destitution and bodily suffering is to cultivate while young all the benevolent and generous feelings of our nature, never by any possibility allowing any opportunity pass of befriending a fellow-traveler, as we are passing along life's journey, for sooner or later the reward will come, the reward of a happy heart and oftentimes a comfortable provision for declining years.

In 1812, a wounded soldier was lying helpless on the plains of Chalmette, a few miles below New-Orleans. A youth passing that way kneeled at his side, inquired as to his wants, conveyed him to a shelter, and remained with him until he was able to leave for his home in the city. Nearly have a century later, the wounded soldier died, but old Judah Touro never forgot the youth who helped him on the battle-field, and left him fifty thousand dollars in money, besides some duties to perform which eventually yielded Mr. Shepherd $100,000 more.

While living in New-Orleans, about the year 1850, a poor young doctor, with a large family and a small practice, often came into my office. He was always courteous, always kind, and always sad; and who could be otherwise when anxiety for to-morrow's bread for wife and children, was always pressing on the heart? But there came a letter one day, with the English post-mark, making inquiries for a young American doctor who had greatly befriended an English gentleman during a long and dangerous attack of sickness in New-Orleans a number of years before. This grateful gentleman had died, and left our poor young doctor a large estate.

Ten years ago and less, there lived in the city of New-York a clergyman whose name and memory are sacred to thousands of grateful, loving, revering hearts. He has not been dead long, he will never die out of the holy affections of the people before whom he came in and went out so many years. Among his people there was one man, and he was of large wealth, who seemed to make it his special business, as it was his highest happiness, to see that his revered pastor wanted nothing. It was not a fitful care. It did not spring up in May, and die long before December came, but through weeks and months and long years it was always the same; incessant, perennial, gushing up alway like a never-failing spring. The pastor died; his loving watcher, by no fault of his own, failed for almost millions; any recovery was absolutely hopeless. The grief that pressed him most was the loss of ability to help the helpless. Men looked on and wondered, and began to question if Providence would let such a man come to want in his gray hairs. But there was an eye upon him. A man of very great wealth said: "He must not suffer who cared so well and faithfully and long for my old minister. He is just the man I want to attend to my estates, and he shall have all he asks for as compensation for his services."

